# Comparative hepatoprotective effects of AMK and PAMK via Nrf2 signaling in broiler breeders

**DOI:** 10.3389/fvets.2025.1672417

**Published:** 2025-09-23

**Authors:** Baili Lu, Jiayu He, Shirou Pan, Junhao Wei, Bingxin Li, Nan Cao, Yunmao Huang, Yunbo Tian, Ngai Cheong, Ying Chen, Danning Xu, Wanyan Li

**Affiliations:** ^1^College of Animal Science and Technology, Zhongkai University of Agriculture and Engineering, Guangzhou, China; ^2^Faculty of Applied Sciences, Macao Polytechnic University, Macao, China; ^3^School of Health Economics and Management, Nanjing University of Chinese Medicine, Nanjing, China

**Keywords:** broiler breeders, oxidative stress, PAMK, traditional Chinese medicine, apoptosis

## Abstract

Atractylodes macrocephala Koidz. (AMK) and its purified polysaccharide fraction (PAMK) are known for their antioxidant, anti-inflammatory, and hepatoprotective properties, showing potential benefits for poultry liver health. This study simulated hepatic oxidative stress in late-laying hens, a physiological stage characterized by increased metabolic demands and reactive oxygen species (ROS) generation, without exogenous hepatotoxic agents. Hens were randomly assigned to three groups: control (basal diet), AMK (basal diet + 15 g/kg AMK), and PAMK (basal diet + 400 mg/kg PAMK). Both AMK and PAMK significantly reduced serum alanine aminotransferase (ALT) and aspartate aminotransferase (AST) activities compared to control. Notably, PAMK showed superior efficacy, decreasing malondialdehyde (MDA) levels by 35.29% vs. 32.87% in AMK, and more effectively increasing antioxidant enzymes superoxide dismutase (SOD) and glutathione peroxidase (GSH-Px). Histopathological analysis revealed better-preserved liver structure and less inflammatory infiltration in PAMK-fed hens. Mechanistically, both treatments upregulated Nrf2 and downstream antioxidant genes, with stronger activation observed in the PAMK group. *In vitro*, PAMK reduced H_2_O_2_-induced ROS accumulation and apoptosis in primary embryonic chicken hepatocytes, effects that were attenuated by the Nrf2 inhibitor ML385. In conclusion, PAMK exerts superior hepatoprotective effects compared to crude AMK by modulating the Nrf2 pathway, mitigating oxidative stress, apoptosis, and autophagy. Future research should evaluate PAMK's long-term safety, synergistic potential with other natural antioxidants, and cost-effectiveness in poultry production.

## 1 Introduction

Broiler breeders, as the foundational stock in modern poultry production, play a crucial role in determining the growth performance and economic efficiency of their offspring. Under intensive genetic selection and high-density rearing systems, broiler breeders exhibit excellent reproductive performance during the peak laying period. However, following the peak, broiler breeders gradually enter a stage characterized by increased metabolic burden and reduced disease resistance. During this period, key metabolic organs, particularly the liver, become more vulnerable to various stressors (1). As the largest metabolic organ in poultry, the liver is not only responsible for nutrient transformation, detoxification, and energy storage, but also plays a pivotal role in regulating oxidative stress and immune responses. Nevertheless, in the post-peak laying period, prolonged high egg production places substantial strain on the liver, often resulting in hepatic dysfunction and exacerbated oxidative damage, which ultimately compromises overall health and sustained reproductive performance (2).

In broiler breeders, hepatic oxidative stress during the post-peak laying period is considered a key factor contributing to functional deterioration. As metabolic demands increase, reactive oxygen species (ROS) accumulate persistently within the body, while the efficiency of the antioxidant defense system, including superoxide dismutase (SOD), catalase (CAT), and glutathione peroxidase (GSH-Px) progressively declines. This imbalance in redox homeostasis renders the liver increasingly susceptible to oxidative damage (3). On one hand, ROS directly attack lipids, proteins, and nucleic acids, leading to structural disruption of organelles and impairment of cellular functions; on the other hand, ROS activate intracellular signaling pathways that further disturb regulatory networks, intensifying the stress response and altering cell fate (4).

The antioxidant system serves as the primary line of defense in maintaining cellular homeostasis. Its dysfunction triggers a cascade of molecular events. Oxidative stress not only causes direct biochemical damage to lipids, proteins, and DNA, but also activates various stress-responsive signaling pathways, thereby inducing apoptosis and autophagy (5). Although apoptosis and autophagy play protective roles under moderate conditions by removing damaged components and preserving homeostasis, excessive or sustained oxidative stress often leads to their simultaneous overactivation. This results in amplified cell death responses, eventually causing irreversible damage to tissue architecture and function. The nuclear factor erythroid 2-related factor 2 (Nrf2) pathway plays a pivotal role in regulating cellular antioxidant responses. Activation of the Nrf2 pathway has been demonstrated to attenuate hepatic oxidative injury caused by various insults, including toxins, metabolic overload, and inflammatory stimuli. Natural compounds such as astaxanthin have been shown to alleviate hepatic lipid metabolic dysregulation by activating Nrf2 signaling (6), highlighting its relevance in hepatoprotection. In addition to Nrf2, the phosphatidylinositol 3-kinase/protein kinase B (PI3K/Akt) signaling cascade has been implicated in oxidative stress regulation and hepatocyte survival. Activation of PI3K/Akt not only promotes antioxidant defenses but also complements Nrf2 activity through cross-talk mechanisms, enhancing cellular resilience against ROS-induced injury. Traditional Chinese decoctions, such as Liuwei Dihuang, have been shown to modulate the PI3K/Akt pathway in the liver, improving insulin sensitivity and reducing oxidative stress in animal models (7), suggesting that herbal formulations may exert multi-pathway hepatoprotective effects.

Given these challenges, nutritional interventions aimed at enhancing hepatic antioxidant capacity and disrupting or delaying the pathological cascade of apoptosis and autophagy have emerged as critical strategies to maintain the health and reproductive performance of broiler breeders during the late laying period. Among these, traditional Chinese medicine (TCM) and its active constituents have garnered increasing attention due to their multi-targeted actions, low toxicity, immunomodulatory effects, and potent antioxidant properties, making them promising candidates as antibiotic substitutes in poultry feed. AMK, a medicinal herb from the Asteraceae family, holds significant medicinal value. Its pharmacological properties encompass a variety of functions, including strengthening the spleen and enhancing vitality, drying dampness and promoting diuresis, as well as arresting sweating and stabilizing pregnancy (8). Modern pharmacological studies have identified various bioactive compounds in AMK, including essential oils, sesquiterpenes, flavonoids, and polysaccharides. Among these, the water-soluble polysaccharide fraction PAMK has received particular attention for its antioxidant, immunomodulatory, anti-inflammatory, and anti-apoptotic properties, highlighting its potential as a functional feed additive (9). Previous studies have demonstrated that the active components of AMK can effectively reduce malondialdehyde (MDA) levels and enhance the body's antioxidant capacity (10). In animal models, PAMK has been shown to significantly increase the activity of antioxidant enzymes, lower hepatic MDA concentrations, and alleviate lipopolysaccharide (LPS)-induced oxidative liver damage (11). Moreover, PAMK can modulate cell fate through multiple mechanisms, including regulation of the BCL2 family (12), downregulation of caspases expression, and suppression of autophagy-related gene expression. These multifaceted protective effects make PAMK a promising natural antioxidant and tissue-protective agent for use in animal health management (13). However, PAMK, as a highly purified active component obtained through multistep extraction from AMK, involves relatively high production costs. In contrast, AMK, which requires simpler processing and is more cost-effective, offers greater practical applicability. Nevertheless, whether AMK can achieve biological effects comparable to those of PAMK remains insufficiently investigated. In addition to containing a certain proportion of crude polysaccharides, AMK also comprises essential oils, flavonoids, and sesquiterpene lactones. Whether these compounds exert synergistic antioxidant, anti-apoptotic, and autophagy-modulating effects warrants further systematic exploration.

We hypothesize that both AMK and PAMK exert hepatoprotective effects during the post-peak laying period by enhancing antioxidant capacity and modulating apoptosis and autophagy through the activation of the Nrf2 pathway, with PAMK exhibiting stronger activity due to its higher purity and targeted action.

## 2 Materials and methods

### 2.1 Animals and treatment

A total of 360 healthy broiler breeders (31 weeks old with similar body weights) were provided by the Gaocun Wen's Breeder Farm, a subsidiary of Wen's Food Group Co., Ltd. Broiler breeders were randomly assigned to three groups: Control, AMK, and PAMK, with 10 replicates per group and 12 broiler breeders per replicate. The Control group was fed a basal diet and managed according to the standard farm immunization schedule. The AMK group received the basal diet supplemented with 15 g/kg AMK. The dosage selection of AMK primarily refers to Veterinary Chinese Materia Medica (edited by Zhong Xiuhui and Liu Zhanmin) and Traditional Chinese Veterinary Medicine (edited by Liu Zhongjie and Xu Jianqin, 3rd edition). The PAMK group received the basal diet supplemented with 400 mg/kg PAMK [the concentration was validated by our research group in previous studies (14, 15)]. The supplementation period lasted for 21 days. The composition and nutritional levels of the basal diet are shown in Table 1, and the experimental treatments are detailed in Table 2.

**Table 1:** Composition and nutrient levels of basal diet (air-dry basis).

**Ingredients**		**Nutrient levels** ^**2**^	
**Items**	**Content (%)**	**Items**	**Content (%)**
Corn	60.55	CP	17.04
Soybean meal	23.90	ME (MJ/kg)	11.77
Limestone	8.00	CF	5.11
Corn gluten meal	2.60	Ca	3.45
Soybean oil	2.35	TP	0.71
CaHPO_4_.2H_2_O	1.60	Lys	0.82
Premix^1^	1.00		
**Total**	**100.00**		

^1^The premix provides the following per kilogram of feed: VA 12,000 IU, VD_3_ 5,000 IU, VB_1_ 3.0 mg, VB_2_ 5.0 mg, VK 2.0 mg, VE 30.0 mg, VB_12_ 1.0 mg, Niacin 30.0 mg, Pantothenic acid 80.0 mg, Folic acid 50.0 mg, Biotin 0.2 mg, Choline 500.0 mg, Fe (FeSO_4_) 100.0 mg, Cu (CuSO_4_.5H_2_O) 80.0 mg, Mn (MnSO_4_) 100.0 mg, I (KI) 0.42 mg, Se (Na_2_SeO_3_) 0.30 mg.

^2^ The metabolizable energy is calculated, while the rest of the values are measured.

**Table 2:** Grouping and processing of experimental animals.

**Group**	**Number**	**Dose of AMK (g/kg)**	**Dose of PAMK (mg/kg)**
Control	120	0	0
PAMK	120	0	400
AMK	120	15	0

Following a 7-day withdrawal period, broiler breeders were euthanized under deep anesthesia by intraperitoneal injection of sodium pentobarbital at a lethal dose (100 mg/kg body weight) to minimize pain and distress. Serum and liver samples were collected immediately, snap-frozen in liquid nitrogen, and stored at –80°C for subsequent analyses. The experimental procedures were approved by the Animal Care and Use Committee of Zhongkai University of Agriculture and Engineering (approval number: ZK202208-19).

### 2.2 Reagents

PAMK (purity ≥95%, catalog number: CY201216) was purchased from Yangling Ciyuan Biotechnology Co., Ltd. Hydrogen peroxide (H_2_O_2_, catalog number: H433859) was obtained from Aladdin Reagent Co., Ltd. (Shanghai, China). Trizol RNA isolation reagent (catalog number: GK20008) and enhanced chemiluminescence (ECL) detection kit (catalog number: GK10008) were purchased from GLPBIO (Montclair, CA, USA). Reverse transcription reagents (catalog number: RR047A) were supplied by Takara Bio Inc. (Japan). PowerUp SYBR Green Master Mix (catalog number: A301-05) was purchased from Kangrun Biotech (Beijing, China). 4% paraformaldehyde (catalog number: BL539A) was obtained from Biosharp (China). The BCA protein assay kit (catalog number: P0010), 5 × SDS-PAGE loading buffer (catalog number: P0015L), primary antibody dilution buffer (catalog number: P00023A), protease inhibitor cocktail (catalog number: ST506), and RIPA lysis buffer (catalog number: P0013B) were purchased from Beyotime Biotechnology (Shanghai, China). HRP-conjugated Goat Anti-Rabbit IgG (H+L, catalog number: SA00001-2) and HRP-conjugated Goat Anti-Mouse IgG (H+L, catalog number: SA00001-1) were purchased from Proteintech (Wuhan, China), along with the following primary antibodies: Beclin 1 Monoclonal Antibody (catalog number: 66665-1-Ig), LC3 Polyclonal Antibody (catalog number: 14600-1-AP), Caspase-9/p35/p10 Polyclonal Antibody (catalog number: 10380-1-AP), Bcl-2 Polyclonal Antibody (catalog number: 26593-1-AP), Caspase-8/p43/p18 Polyclonal Antibody (catalog number: 13423-1-AP), NQO1 Polyclonal Antibody (catalog number: 11451-1-AP), HO-1/HMOX1 Polyclonal Antibody (catalog number: 10701-1-AP), ATG5 Polyclonal Antibody (catalog number: 10181-2-AP), KEAP1 Polyclonal Antibody (catalog number: 10503-2-AP), BAX Polyclonal Antibody (catalog number: 50599-2-Ig), BAK Polyclonal Antibody (catalog number: 29552-1-AP), and Nrf2/NFE2L2 Polyclonal Antibody (catalog number: 16396-1-AP). Collagenase IV (catalog number: 2091MG100) was purchased from Biofrox (Germany), and DMEM (catalog number: C11995500CP) was obtained from GIBCO (Thermo Fisher Scientific, USA).

### 2.3 AMK component analysis

The AMK was purchased from Xiaoguangyan Herbal Medicine Store in Liwan District, Guangzhou, with its origin in Zhejiang Province. The AMK was air-dried, ground into a fine powder using a high-speed grinder, and then sifted through a 60-mesh sieve to obtain the AMK powder. The content of the major components of AMK was analyzed and determined by Guangzhou Huibiao Checking & Measuring Technology Center.

### 2.4 Histopathological staining

Liver was fixed in 4% paraformaldehyde for 48 h, followed by paraffin embedding. Sections were cut at a thickness of 5 μm. Deparaffinization was performed using xylene, followed by gradual rehydration through a series of ethanol solutions and rinsing with water. For hematoxylin and eosin (H&E) staining, sections were immersed in Harris hematoxylin solution for 15 min, rinsed with water, and then differentiated using 1% acid ethanol and 0.6% ammonia water. Subsequently, sections were stained with eosin for 3 min. Finally, the sections were dehydrated, coverslipped, and microscopic images were acquired for analysis. This procedure was assisted by Hubei Bios Biotechnology Co., Ltd.

### 2.5 Transmission electron microscopy

Approximately 3 mm^2^ of liver from meat-type chickens was excised and fixed in 2.5% glutaraldehyde solution at 4°C. Tissue sampling was performed rapidly within 1 min to minimize mechanical damage, and tissue blocks were controlled to an area within 3 mm^2^. After washing with PBS, the tissue orientation was marked, then immediately immersed in electron microscopy fixative and fixed at room temperature for 2 h before transferring to 4°C for storage. Samples were rinsed three times in 0.1 M phosphate buffer (pH 7.4) for 15 min each, followed by fixation in 1% osmium tetroxide at room temperature in the dark for 2 h. After washing with phosphate buffer, samples were dehydrated through a graded ethanol series from 30% to 100% (15 min each), then treated with isopentyl acetate for 15 min. After drying, specimens were mounted on conductive carbon tape attached to a stub, sputter-coated with gold for 30 s using an ion sputter coater, and examined under a transmission electron microscope for imaging. Preparation of TEM specimens was assisted by Guangzhou KingMed Diagnostics Center.

### 2.6 TUNEL assay for apoptosis detection

Approximately 3 mm^2^ of liver from meat-type chickens was excised and fixed in 4% paraformaldehyde. Sections were prepared following the steps below. Paraffin-embedded sections were deparaffinized through xylene and a graded ethanol series to water, followed by proteinase K digestion at 37°C for 22 min and rinsed with PBS. Next, permeabilization was performed by incubating sections at room temperature for 20 min in 0.1% Triton X-100 permeabilization buffer, then rinsed again with PBS. After equilibration at room temperature, sections were incubated with the reaction mixture containing TDT, dUTP, and buffer at 37°C in a humidified chamber for 2 h. Cell nuclei were counterstained with DAPI at room temperature in the dark for 10 min, rinsed with PBS, and coverslipped. Slides were observed and imaged under a fluorescence microscope. DAPI staining showed nuclei in blue, while FITC-labeled apoptotic nuclei fluoresced green. Apoptotic rates were calculated using ImageJ software version 1.5.2.

### 2.7 Hepatocyte isolation and culture

Twelve-day-old Specific Pathogen Free (SPF) chicken embryos were selected, confirmed to be free of lesions, and their surfaces disinfected with 75% ethanol. PAMK solution (60 μg/mL), liver wash solution (PBS containing 2% penicillin-streptomycin), and liver digestion solution (0.02% type IV collagenase in PBS) were prepared. The embryos were euthanized, and the abdomen was opened to excise the liver. Fascia was removed, and the liver tissues were placed into 50 mL centrifuge tubes. Collagenase digestion solution was added, and the tissue was gently pipetted and incubated in a water bath for digestion. The cell suspensions were repeatedly centrifuged and washed to remove red blood cells, then resuspended in DMEM medium. The cell concentration was adjusted to 1 × 10^6^ cells/mL and seeded for culture. After 24 h of incubation, the culture medium was discarded, cells were washed with PBS, and fresh medium was added to continueculture.

### 2.8 Establishment of oxidative damage model

Due to potential impurities in AMK that may affect cell growth, AMK was unsuitable for cell culture experiments due to potential impurities affecting cell growth. Therefore, only PAMK extracted through advanced processing was used for *in vitro* cell assays. Preliminary experiments were conducted to assess the effects of different concentrations of PAMK and H_2_O_2_ on hepatocytes by measuring the levels of ROS (Supplementary Figure S1). Based on these results, 60 μg/mL PAMK was selected for pre-treatment of chicken embryo hepatocytes for 24 h, followed by exposure to 0.6 mmol/L H_2_O_2_ for 24 h to establish an oxidative damage model. This model was designed to simulate oxidative stress and evaluate the protective effects and potential mechanisms of PAMK against oxidative injury, providing a reliable experimental foundation for further molecular studies.

For model construction and treatment, chicken embryo hepatocytes were cultured at 39°C in a 5% CO_2_ incubator. Cell morphology and status were monitored under an inverted microscope, and culture medium was replaced every 24 hours. When cells reached good condition with an adhesion rate above 80%, they were divided into four groups: Control (C), PAMK alone (P), PAMK plus H_2_O_2_ (P+H), and H_2_O_2_ alone (H), as detailed in Table 3. After 24 h of drug treatment, cells were collected for subsequent assays.

**Table 3:** Grouping and treatment of oxidative damage model.

**Group**	**Dose of PAMK (μg/mL)**	**Dose of H_2_O_2_ (mmol/L)**
C	0	0
P	60	0
P+H	60	0.6
H	0	0.6

### 2.9 Construction of Nrf2 inhibition model

To further investigate whether PAMK alleviates oxidative stress-induced hepatocyte injury by modulating Nrf2, an ML385-induced Nrf2 inhibition model was established. Chicken embryo hepatocytes were cultured at 39°C with 5% CO_2_. Cell morphology and condition were monitored under an inverted microscope. When cells reached a healthy state with over 80% adhesion, the original culture medium was discarded, and cells were washed twice with PBS to remove residual medium. Then, culture medium containing 60 μg/mL PAMK was added, and cells were incubated for 20 h at 39°C and 5% CO_2_ to ensure adequate drug exposure. Subsequently, 2 μM ML385 was added, and co-incubation continued for 4 h. Cells were divided into four groups: Control (C), ML385 alone (M), PAMK alone (P), and PAMK plus ML385 (P+M), as detailed in Table 4. After treatment, cells were collected for subsequent analysis of relevant indicators.

**Table 4:** Grouping and treatment of Nrf2 inhibition model.

**Group**	**Dose of PAMK (μg/mL)**	**Dose of ML385 (μM)**
C	0	0
M	0	2
P	60	0
P+M	60	2

### 2.10 Detection of ROS in primary chicken embryo hepatocytes

When chicken embryo hepatocytes reached over 80% confluency, the culture medium was discarded, and cells were washed three times with PBS to remove cell debris and serum. Under light-protected conditions, the ROS-sensitive fluorescent probe DCFH-DA was diluted 1:500 in serum-free medium and mixed thoroughly. This staining solution was applied to the cells and incubated at 37°C for 30 min, with gentle shaking every 10 min. After incubation, cells were observed and imaged using a fluorescence microscope. The percentage of ROS-positive cells was calculated as:


(1) $$\begin{array}{c} \text{Positive cell rate (\%)}=\frac{\text{Number of green fluorescent cells}}{\text{Total number of cells}}\times 100\% \end{array}$$

### 2.11 Real-time quantitative PCR

Total RNA was extracted from liver tissue and primary chicken embryo hepatocytes using the Trizol method. Reverse transcription was performed according to the instructions provided in the Takara kit. Target gene sequences were obtained from the NCBI database, and primers were designed and synthesized by Youkang Biotechnology Co., Ltd. (Guangzhou, China). The sequences of the primers used in this study are listed in Table 5.

**Table 5:** Primer sequences.

**Gene**	**Accession number**	**Primer sequences (5'-3')**
*β-actin*	NM_205518.2	F: CTGACTGACCGCGTTACTCC
		R: CATACCAACCATCACACCCTGA
*BCL2*	XM_046910476.1	F: GATCGTCGCCTTCTTCGAGT
		R: ATTCCACAAAGGCATCCGGT
*BAK*	NM_001030920.3	F: TATTCGCTTCCTTCCCCTGCGG
		R: CCACCTGGTCCTCTGCATTG
*BAX*	XM_040654813.2	F: GAGTGGGGAGCGTTCGAG
		R: GGTGGAGGCTGAGATATGGG
*Caspase8*	NM_204592.4	F: TGTGGCAAAGTGGACAAGAG
		R: AGCCAAATGGCACTGTCTTC
*Caspase9*	XM_046931415.1	F: GCCTGTGGAGGAGAACAAAAG
		R: GTCTGGCTCGTCCTCATTCC
*Beclin1*	XM_015299595.4	F: TACGCAGGTCAGCTTTGTGT
		R: GTGAAGGCCTCCTCGCTC
*ATG5*	XM_046914035.1	F: GAATATGAAGGTACGCCACTGA
		R: CTCCAAGGAAGGGCTGTATTT
*LC3-I*	XM_040688401.2	F: GCATCCAAACAAAATCCCAGTC
		R: AAGCCATCCTCATCCTTCTCCT
*LC3-Π*	NM_001031461.2	F: CTTCTTCCTCCTGGTGAACG
		R: GCACTCCGAAAGTCTCCTGA
*mTOR*	XM_040689168.2	F: AGGAGGAGTCCACCAGGTTT
		R: GGTGGCGTTACCTCCTTCAA
*MsrA*	XM_046939237.1	F: CACCTTCAGGATGGGCGAC
		R: CCCAGAAACAGCCCATACCAAA
*MsrB*	NM_001135558.3	F: AGGCGAAGTGTTCAAGGACC
		R: CCTGCGGGCTTTTGCCTTTAG
*Nrf2*	NM_001396902.1	F: CTTTCTCAGGGTTTCTTTGCTTT
		R: TCAATCAAGTTCATGTCCAAAAAGG
*HMOX1*	NM_205344.2	F: AAACTTCGCAGCCACACAAC
		R: GACCAGCTTGAACTCGTGGA
*NQO1*	NM_001277620.2	F: AACCTCTTTCAACCACGCCA
		R: TTCTTGAGGGGTCCGGTGAT
*Keap1*	XM_015288374.4	F: GCTGGAGGAGATGGAGAAGG
		R: TCCTGGTGATGGTGATGAGG

The reaction system for qRT-PCR was prepared according to the manufacturer's instructions, as shown in Table 6. Quantitative PCR was conducted using the QuantStudio 7 Real-Time PCR System with the following cycling parameters: 50°C for 2 min, 95°C for 2 min, followed by 40 cycles of 95°C for 15 s and 60°C for 30 s. The housekeeping gene β-actin was used as the internal reference, and the relative mRNA expression levels of target genes were calculated using the 2^−Δ^^Δ^^*Ct*^ method.

**Table 6:** RT-qPCR reaction system.

**Component**	**Volume (μL)**
cDNA	0.5
2 × Power Up^TM^ SYBR^TM^ Green Master Mix	5
F Primer (10 μM)	0.25
R Primer (10 μM)	0.25
DEPC	4

### 2.12 Western blot

Phosphatase inhibitor, EDTA, and protease inhibitor were mixed with RIPA lysis buffer at a ratio of 1:1:1:50. Approximately 0.1 g of liver tissue was placed in a 2 mL centrifuge tube, to which 1 mL of the mixed lysis buffer and two stainless steel beads were added. The tissue was homogenized using a tissue grinder. For primary chicken embryo hepatocytes, cells were lysed with the same buffer, scraped into centrifuge tubes, and centrifuged at 12,000 rpm for 15 min at 4°C. After discarding the supernatant, the pellet was further lysed using an ultrasonic cell disruptor. Protein concentration was determined using a BCA protein assay kit. Protein samples were mixed with SDS-PAGE loading buffer at a 4:1 ratio and denatured by heating at 100°C for 5 min, then stored at –80°C.

Proteins were separated via SDS-PAGE and transferred onto PVDF membranes using a wet transfer method. Membranes were blocked with 5% non-fat dry milk for 2 h at room temperature. Primary antibodies were diluted according to the manufacturer's instructions and incubated with the membranes overnight at 4°C. After washing three times with PBST (5–10 min each), membranes were incubated with secondary antibodies at room temperature for 1 h, followed by three additional PBST washes. Signals were visualized and imaged. Band intensities were analyzed using ImageJ software, and the relative expression levels of the target proteins were normalized to β-actin.

### 2.13 Data processing and statistical analysis

All data were statistically analyzed using GraphPad Prism 10.0 software. Differences among independent samples were assessed by one-way ANOVA followed by Tukey's multiple comparison test. Data are presented as means ± standard deviation (S.D.). In bar charts, different lowercase letters indicate statistically significant differences between groups (*P* < 0.05), whereas the same letters indicate no significant difference (*P* > 0.05).

## 3 Result

### 3.1 AMK component analysis

As shown in Table 7, the main nutritional components of AMK include moisture, crude protein, calcium, total phosphorus, and crude polysaccharides.

**Table 7:** Analysis of AMK components.

**Item**	**Component**
Moisture	8.0%
Crude protein	9.42%
Calcium	0.665%
Total phosphorus	0.27%
Crude polysaccharides	14.84 g/100g

### 3.2 Effects of AMK and PAMK on egg weight and egg production rate

**Figure 1:**
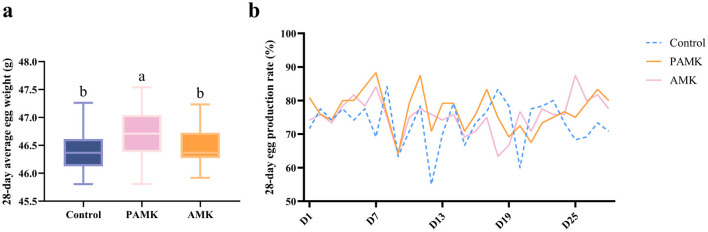
Effects of AMK and PAMK on egg weight and egg production rate. **(a)** 28-day average egg weight; **(b)** 28-day egg production rate. Different letters indicate significant differences (*P* < 0.05).

As shown in Figure 1a, over the 28-day period, the average egg weight of the PAMK group was significantly higher than that of the Control and AMK groups (*P* < 0.05). In contrast, there was no significant difference in average egg weight between the AMK group and the Control group (*P* > 0.05). As shown in Figure 1b, the egg production rate of the Control group exhibited a more fluctuating trend over the 28 days, whereas the PAMK and AMK groups showed a relatively stable trend, maintaining a high level with only slight fluctuations.

### 3.3 Effects of AMK and PAMK on liver histomorphology in broiler breeders

**Figure 2:**
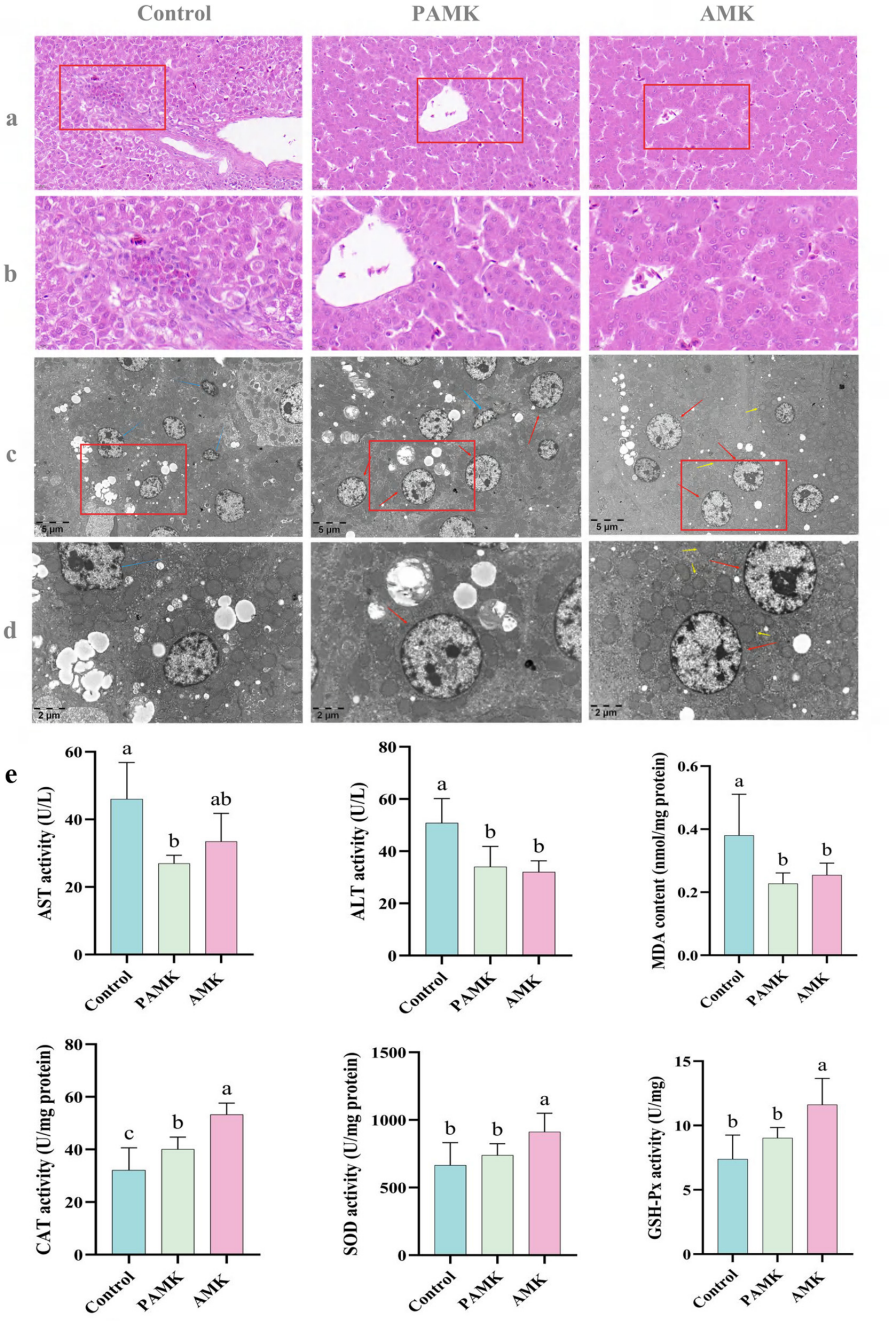
Protective effects of AMK and PAMK on the liver of broiler breeders. **(a)** Liver H&E staining at 400x magnification; **(b)** liver H&E staining at 800x magnification; **(c)** Liver tissue transmission electron microscopy (TEM) at 5,000x magnification; **(d)** liver tissue TEM at 12,000x magnification. Red arrows indicate normal hepatocytes, blue arrows indicate shrunken hepatocytes, and yellow arrows indicate mitochondria; **(e)** serum activities of AST and ALT, and hepatic tissue levels of malondialdehyde (MDA), catalase (CAT), superoxide dismutase (SOD), and glutathione peroxidase (GSH-Px). Data are presented as mean ± S.D. (*n* = 7). Different letters indicate significant differences (*P* < 0.05).

As shown in Figures 2a, b, the liver in the Control group exhibited generally normal architecture, though mild disorganization of hepatic cords and reduced cytoplasmic staining in hepatocytes were observed. Scattered small inflammatory foci were occasionally present within the hepatic parenchyma. In addition, eosinophilic granulocyte infiltration and hepatocellular necrosis were noted around the portal area, with fibrosis surrounding larger central veins. In contrast, liver tissues from both the AMK and PAMK groups demonstrated normal structural integrity, with well-arranged hepatic cords and intact hepatocyte morphology, although mild sinusoidal dilation was occasionally observed.

As illustrated in Figures 2c, d, hepatocytes in the Control group exhibited marked pathological alterations, including mitochondrial swelling and vacuolization, peripheral chromatin condensation, irregular cell contours, and cellular shrinkage—hallmarks of early-stage apoptosis. In contrast, hepatocytes in both the AMK and PAMK groups maintained relatively normal ultrastructural integrity, characterized by abundant cytoplasmic glycogen and lipid droplets, with no apparent ultrastructural abnormalities. These findings suggest that dietary supplementation with AMK or PAMK confers hepatoprotective effects, potentially alleviating hepatocellular apoptosis induced by the metabolic burden of peak egg production.

### 3.4 Effects of AMK and PAMK on liver function and hepatic antioxidant capacity in broiler breeders

As shown in Figure 2e, both AMK and PAMK supplementation significantly reduced serum levels of ALT and AST compared to the Control group (*P* < 0.05). These results indicate that dietary supplementation with AMK or PAMK does not impair hepatic function and can effectively alleviate liver cell damage, thereby helping to maintain or improve liver function. Furthermore, compared with the Control group, the AMK group showed significantly increased activities of CAT, SOD, and GSH-Px (*P* < 0.05), and a significant reduction in MDA levels (*P* < 0.05). The PAMK group also exhibited a significant decrease in hepatic MDA content (*P* < 0.05) along with enhanced CAT activity (*P* < 0.05). These findings suggest that dietary supplementation with AMK or PAMK can enhance the hepatic antioxidant defense system in broiler breeder hens to a certain extent.

### 3.5 Effects of AMK and PAMK on hepatocyte apoptosis in broiler breeders

**Figure 3:**
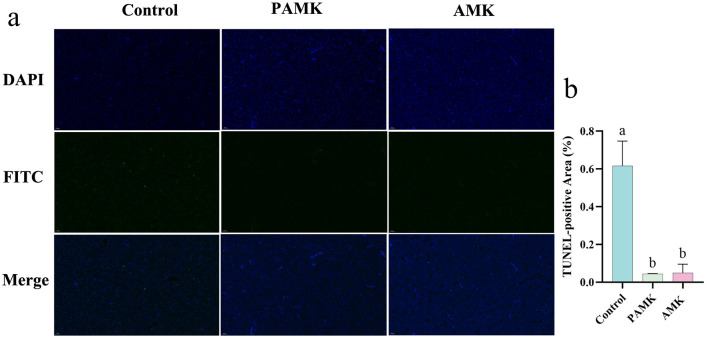
Effects of AMK and PAMK on hepatocyte apoptosis in broiler breeders. **(a)** TUNEL staining of liver tissue (800x magnification); **(b)** quantification of apoptotic cells detected by TUNEL assay in liver. Data are presented as mean ± S.D. (*n* = 3). Different letters indicate significant differences (*P* < 0.05).

To investigate the effects of AMK and PAMK on hepatocyte apoptosis in the liver of broiler breeder, TUNEL staining was performed. As shown in Figures 3a, b compared with the Control group, the number of TUNEL-positive apoptotic cells in the liver was significantly decreased in both the AMK and PAMK groups (*P* < 0.05). These results indicate that dietary supplementation with AMK or PAMK effectively inhibits hepatocyte apoptosis in broiler breeders.

### 3.6 Effects of AMK and PAMK on antioxidant capacity in the liver of broiler breeders

**Figure 4:**
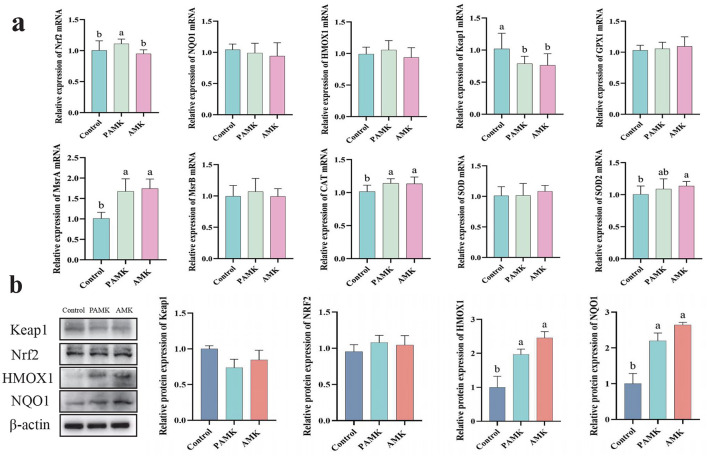
Effects of AMK and PAMK on antioxidant capacity in the liver of broiler breeders. **(a)** Relative mRNA expression of antioxidant-related genes detected by qPCR (*n* = 7); **(b)** protein expression of antioxidant-related proteins detected by Western blot (*n* = 3). Data are presented as mean ± S.D. Different letters indicate significant differences (*P* < 0.05).

As shown in Figure 4a, compared with the Control group, the PAMK group exhibited a significant increase in the relative mRNA expression levels of Nrf2, MrsA, and CAT, while the relative mRNA expression level of Keap1 was markedly decreased (*P* < 0.05). Similarly, the AMK group showed a significant upregulation of MrsA, CAT, and SOD2 mRNA expression, accompanied by a significant reduction in Keap1 mRNA expression (*P* < 0.05). As illustrated in Figure 4b, the protein expression levels of HMOX1 and NQO1 were significantly elevated in both PAMK and AMK groups (*P* < 0.05). These results indicate that dietary supplementation with PAMK or AMK can enhance the antioxidant capacity of broiler chicken liver and effectively protect the liver from oxidative damage.

### 3.7 Effects of AMK and PAMK on apoptosis and autophagy in liver of broiler breeders

**Figure 5:**
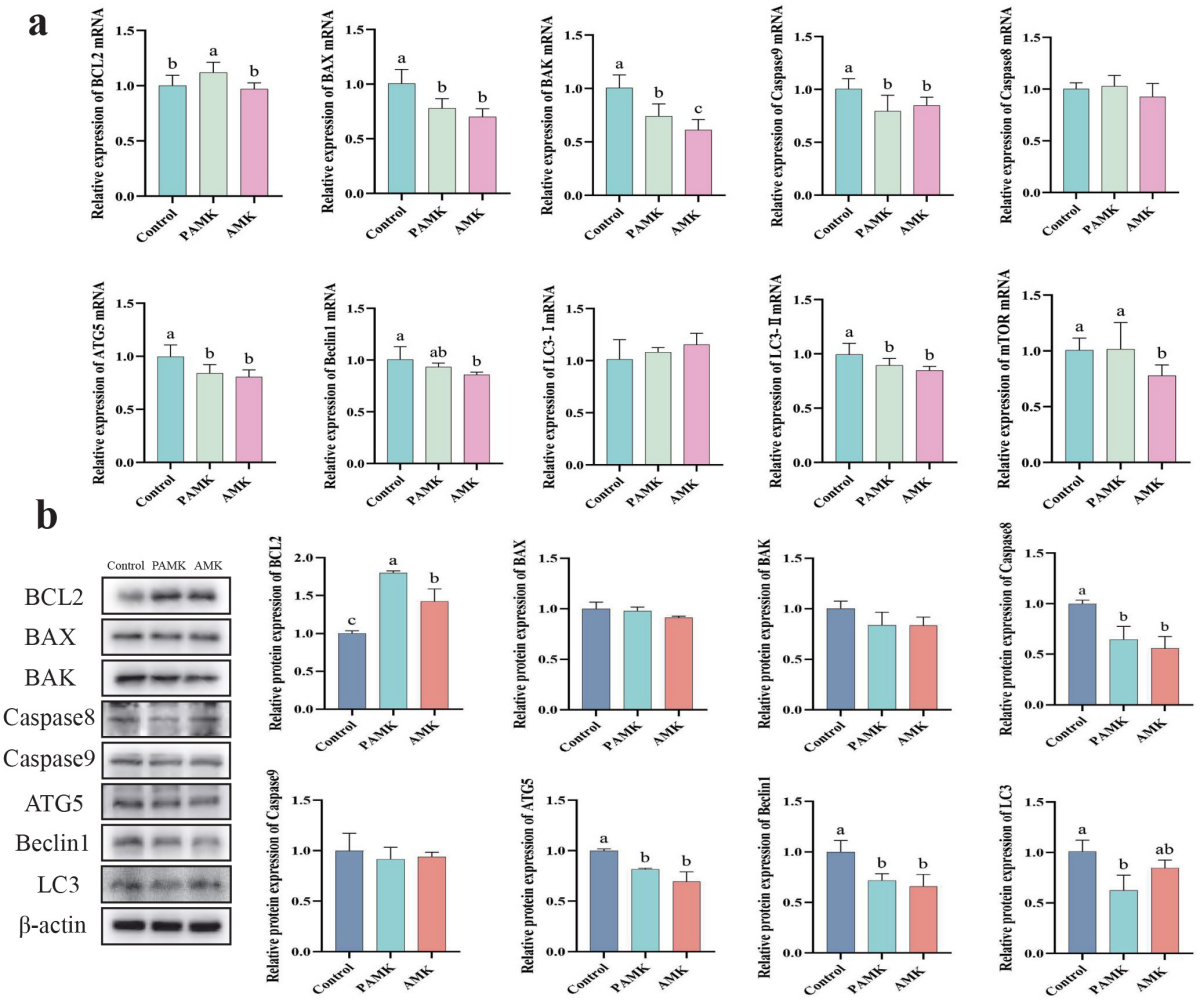
Effects of AMK and PAMK on apoptosis and autophagy in liver of broiler breeders. **(a)** Relative mRNA expression of apoptosis- and autophagy-related genes detected by qPCR (*n* = 7); **(b)** protein expression of apoptosis- and autophagy-related proteins detected by Western blot (*n* = 3). Data are presented as mean ± S.D. Different letters indicate significant differences (*P* < 0.05).

As shown in Figure 5a, compared with the Control group, the PAMK group exhibited a significant increase in the relative mRNA expression levels of BCL2 and mTOR (*P* < 0.05). Both PAMK and AMK groups showed significant decreases in the relative mRNA expression levels of BAX, BAK, Caspase9, ATG5, and LC3-II (*P* < 0.05), while the AMK group substantially downregulated Beclin1 mRNA expression (*P* < 0.05). To further verify gene expression at the protein level, Western blot analysis was performed. As shown in Figure 5b, the results demonstrated that the relative protein expression of BCL2 in both PAMK and AMK groups was significantly higher than that in the Control group (*P* < 0.05). Meanwhile, the relative protein expression levels of Caspase8, ATG5, and Beclin1 were statistically lower in the PAMK and AMK groups compared to Control, consistent with the mRNA expression trends. These findings further confirm the involvement of post-transcriptional regulation. Collectively, the consistent results from Western blot and mRNA analyses suggest that these genes, through modulating the expression of proteins such as BCL2 and mTOR, may play a crucial role in the regulation of apoptosis and autophagy in broiler breeders liver.

### 3.8 Effects of PAMK on ROS levels in oxidatively damaged chicken embryo hepatocytes

**Figure 6:**
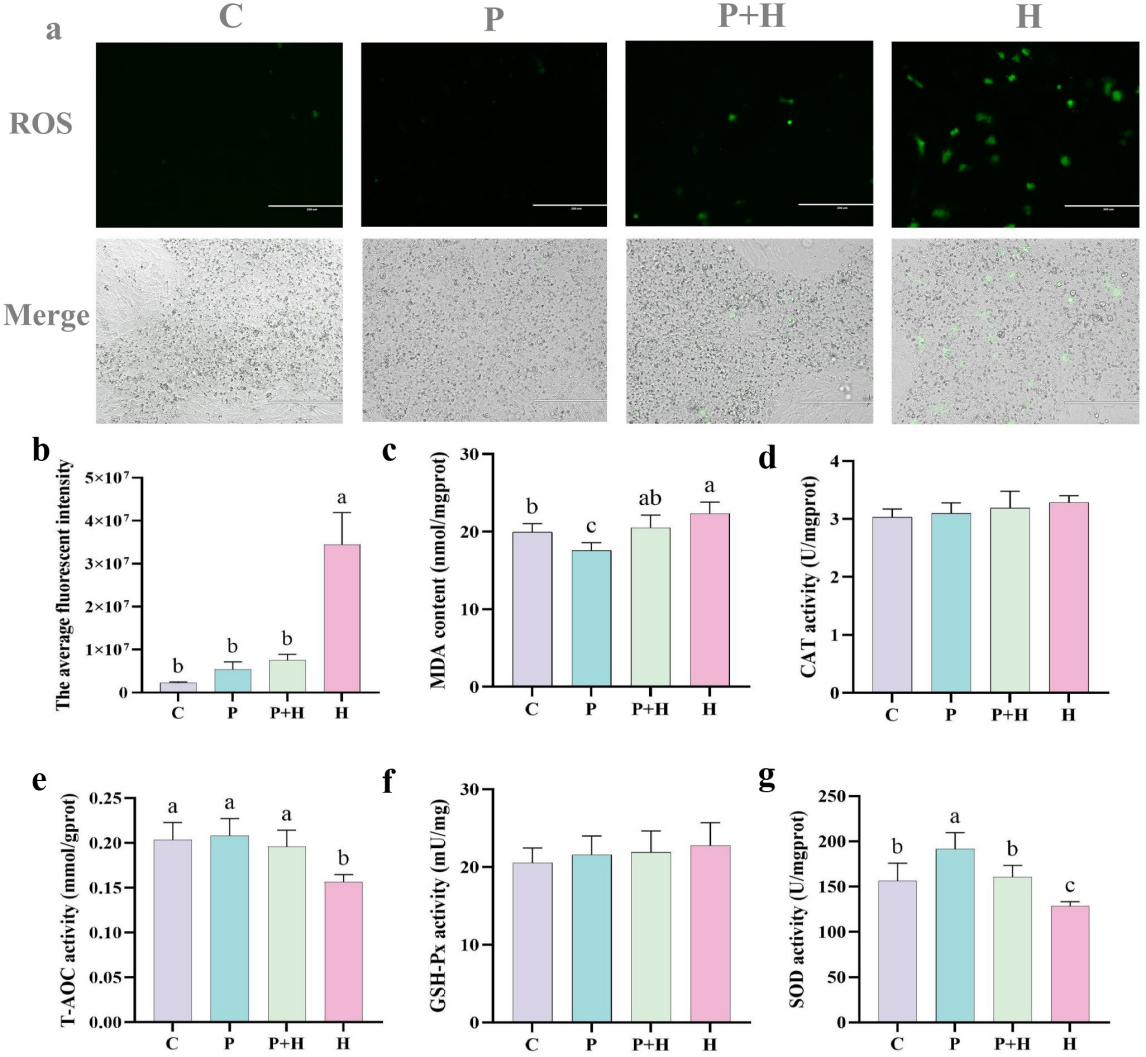
PAMK alleviates H_2_O_2_-induced oxidative damage. **(a)** Fluorescence images of ROS in chick embryonic hepatocytes (200x) (*n* = 3); **(b)** quantification of average fluorescence intensity; **(c)** MDA content; **(d)** CAT activity; **(e)** T-AOC; **(f)** GSH-Px activity; **(g)** SOD activity. Data are presented as mean ± S.D. (*n* = 7). Different letters indicate significant differences (*P* < 0.05).

Chick embryonic hepatocytes were co-cultured with PAMK and H_2_O_2_ for 24 h, after which the cells were collected for ROS detection. As shown in Figures 6a, b, compared to the C group, the H group exhibited a significant increase in the number of ROS-positive cells (*P* < 0.05), while the P group showed no significant difference (*P* > 0.05). Compared with the H group, the P+H group showed a significant reduction in ROS-positive cells (*P* < 0.05). These results indicate that PAMK effectively alleviates H_2_O_2_-induced oxidative damage by substantially decreasing ROS levels, thereby reducing oxidative stress-induced hepatocyte injury and exerting a protective effect on liver cells.

To further verify the antioxidant effect of PAMK on hepatocytes, related antioxidant parameters were measured in chick embryonic hepatocytes. As shown in Figure 6c, compared with the C group, the H group showed a significant increase in MDA content (*P* < 0.05), whereas the P+H group significantly decreased MDA levels. Moreover, as shown in Figures 6e, g, the H group showed a significant decrease in total antioxidant capacity (T-AOC) and SOD activity (*P* < 0.05), while the P+H group considerably enhanced both T-AOC and SOD activities. However, as shown in Figures 6d, f, there were no significant differences in CAT and GSH-Px among different groups in hepatocytes (P > 0.05). These findings demonstrate that PAMK enhances antioxidant enzyme activities and overall antioxidant capacity in hepatocytes, alleviating H_2_O_2_-induced oxidative damage and playing an active role in maintaining redox homeostasis in chicken embryo hepatocytes.

### 3.9 Effect of PAMK on antioxidant capacity in hepatocytes under oxidative stress

**Figure 7:**
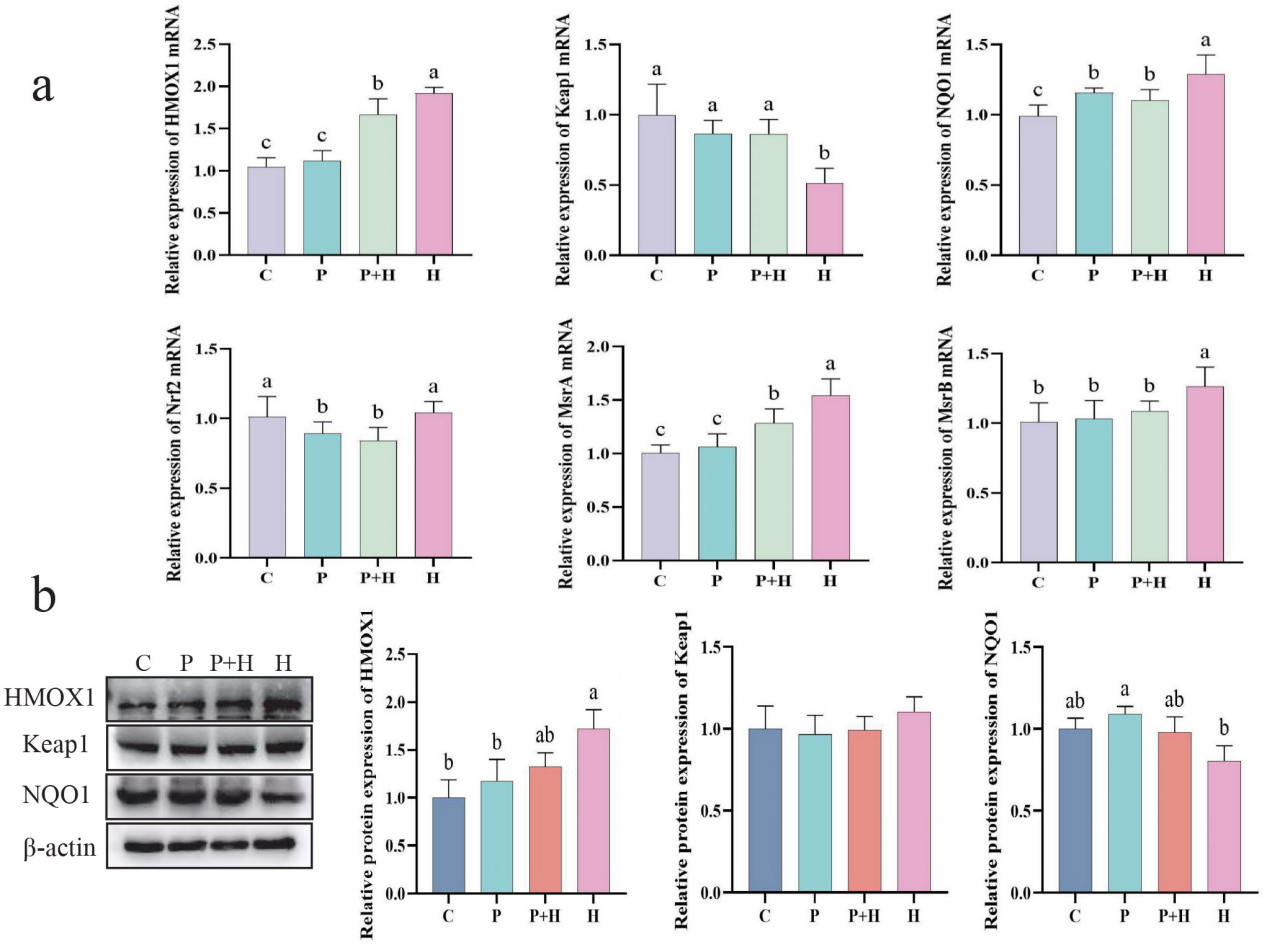
Effect of PAMK on antioxidant capacity in hepatocytes under oxidative stress. **(a)** mRNA expression levels of antioxidant-related genes detected by qPCR (*n* = 5); **(b)** Protein expression levels of antioxidant-related genes detected by Western blot (*n* = 3). Data are presented as mean ± S.D. Different letters indicate significant differences (*P* < 0.05).

In the oxidative stress model, we confirmed the generation of ROS and its impact on cells, as well as changes in related antioxidant indicators. To further investigate the underlying antioxidant mechanisms, we examined the mRNA expression and protein levels of antioxidant-related genes to explore their regulatory roles during oxidative stress. As shown in Figure 7a, compared with the C group, the H group exhibited considerably increased relative mRNA expression of HMOX1, NQO1, MsrA, and MsrB, alongside a significant decrease in Keap1 mRNA expression (*P* < 0.05). Compared with the H group, the P+H group showed significantly reduced mRNA expression levels of HMOX1, NQO1, Nrf2, MsrA, and MsrB (*P* < 0.05), and a significant increase in Keap1 mRNA expression (*P* < 0.05). As shown in Figure 7b, compared with the control group, the H group demonstrated a significant increase in HMOX1 protein expression (*P* < 0.05) and a slight decrease in NQO1 protein expression (*P* > 0.05), while the P+H group showed no significant differences in HMOX1, Keap1, and NQO1 protein levels compared with the C group (*P* > 0.05). These findings indicate that oxidative stress enhances cellular antioxidant capacity by upregulating antioxidant gene expression, whereas PAMK may attenuate the cellular protective response by interfering with the antioxidant reaction. Therefore, these results are consistent with the expected outcomes of the oxidative stress model and further confirm the critical role of antioxidant genes and their regulatory mechanisms in the oxidative stress response.

### 3.10 Effect of PAMK on apoptosis and autophagy in hepatocytes under oxidative stress

**Figure 8:**
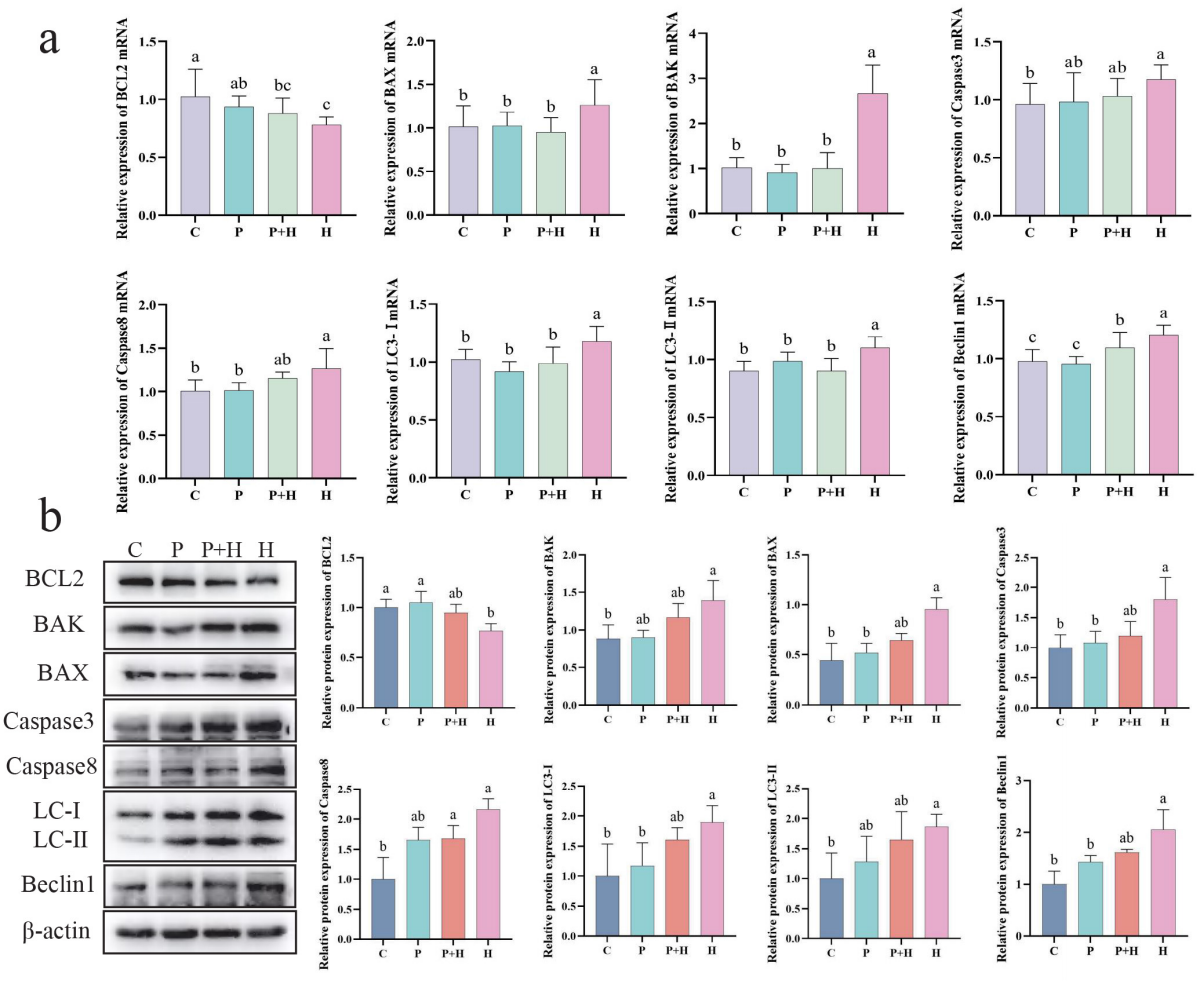
Effect of PAMK on apoptosis and autophagy in hepatocytes under oxidative stress. **(a)** mRNA expression levels of apoptosis- and autophagy-related genes detected by qPCR (*n* = 5); **(b)** protein expression levels of apoptosis- and autophagy-related genes detected by Western blot (*n* = 3). Data are presented as mean ± S.D. Different letters indicate significant differences (*P* < 0.05).

Oxidative stress not only challenges the antioxidant system but also induces apoptosis and autophagy in cells. As shown in Figure 8a, compared with the C group, the H group exhibited a significant decrease in BCL2 mRNA expression (*P* < 0.05), and significant increases in BAX, BAK, Caspase3, Caspase8, LC3-I, LC3-II, and Beclin1 mRNA expression levels (*P* < 0.05). Compared with the H group, the P+H group showed significant reductions in BAX, BAK, LC3-I, LC3-II, and Beclin1 mRNA levels (*P* < 0.05). As shown in Figure 8b, compared with the C group, the H group exhibited a significant downregulation of BCL2 protein expression (*P* < 0.05) and significant upregulation of BAX, BAK, Caspase3, Caspase8, LC3-I, LC3-II, and Beclin1 protein expression (*P* < 0.05), whereas the P+H group partially restored the expression levels of apoptosis- and autophagy-related proteins. In summary, oxidative stress plays a critical role in regulating cell death and autophagy, and PAMK may alleviate the detrimental effects of oxidative stress by modulating the expression of key proteins involved in these processes.

### 3.11 Relationship between the antioxidant effect of PAMK and the Nrf2 pathway

**Figure 9:**
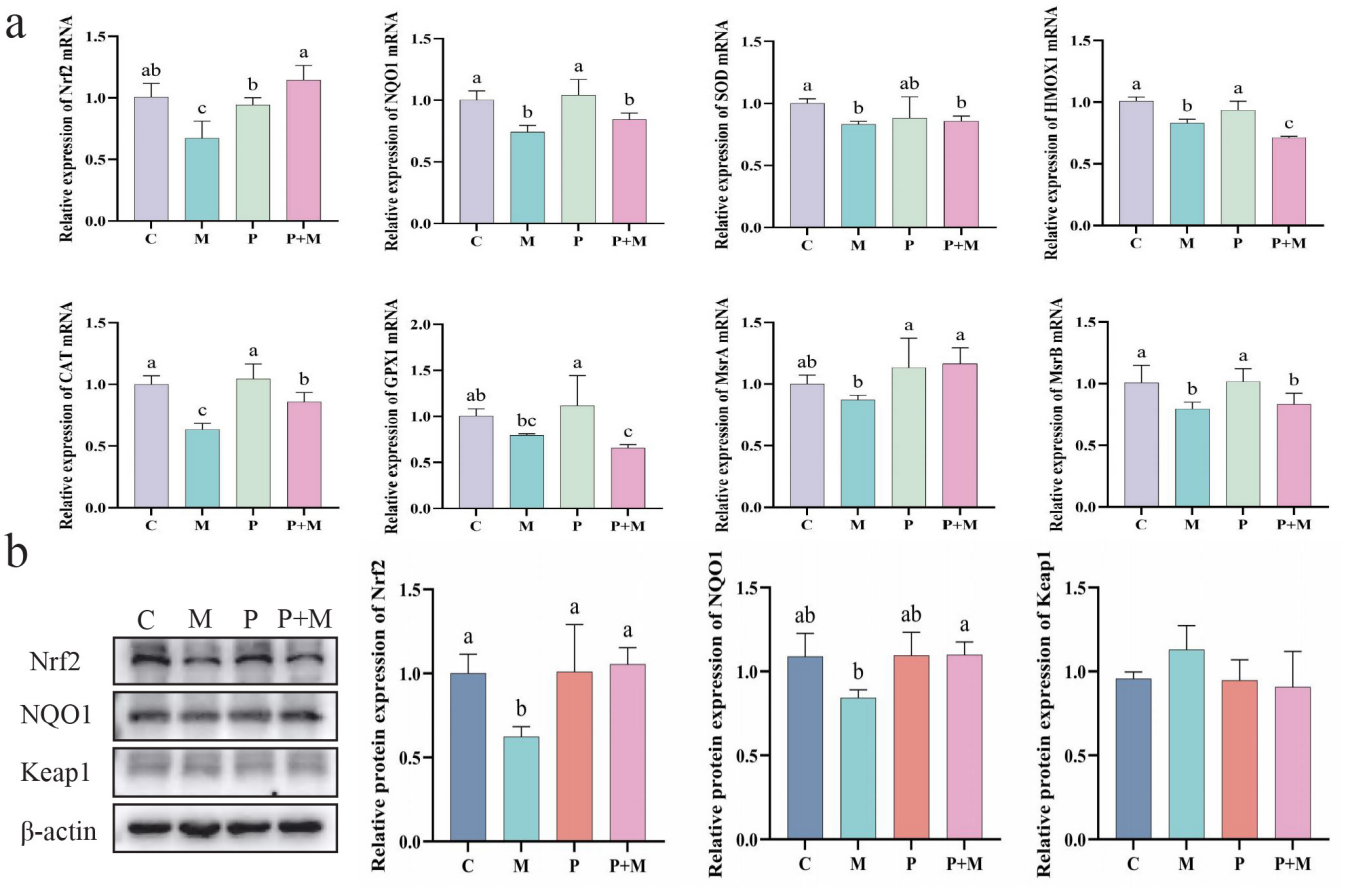
Relationship between the antioxidant effect of PAMK and the Nrf2 pathway. **(a)** mRNA expression of antioxidant-related genes detected by qPCR (*n* = 5); **(b)** Protein expression of antioxidant-related genes detected by Western blot (*n* = 3). Data are presented as mean ± S.D. Different letters indicate significant differences (*P* < 0.05).

To further investigate whether the antioxidant effect of PAMK in alleviating oxidative stress is related to the Nrf2 signaling pathway, ML385 was used to establish an oxidative stress model in chicken embryonic hepatocytes. As shown in Figure 9a, compared with the C group, the M group exhibited a significant decrease in the mRNA expression levels of Nrf2, NQO1, SOD, HMOX1, CAT, and MsrB (*P* < 0.05). Compared with the M group, the P+M group showed significantly increased mRNA expression of Nrf2, CAT, and MsrA (*P* < 0.05). As shown in Figure 9b, the M group exhibited a significant downregulation of Nrf2 protein expression compared with the C group (*P* < 0.05), along with a reduction in NQO1 protein levels. In contrast, the P+M group markedly upregulated Nrf2 and NQO1 protein expression (*P* < 0.05), consistent with the mRNA results. Overall, these findings suggest that the protective effect of PAMK against oxidative stress may be closely associated with the regulation of the Nrf2 pathway, acting to restore the expression of Nrf2 and its downstream antioxidant genes, thereby mitigating oxidative damage.

## 4 Discussion

During the late laying period, broiler chickens are highly susceptible to hepatic oxidative stress, which impairs antioxidant defense mechanisms, triggers apoptosis and autophagy, and leads to deteriorated liver function (16, 17). In our study, we found that dietary supplementation with PAMK significantly alleviated oxidative stress-induced hepatocellular injury, both *in vivo* and *in vitro*. These protective effects are associated with the regulation of redox balance, inhibition of cell death pathways, and activation of the Keap1/Nrf2 signaling cascade, a key mechanism underlying antioxidant defense.

Oxidative stress, as a causative factor, not only directly damages cellular membrane lipids and mitochondrial structures but also activates multiple signaling pathways that induce programmed cell death, particularly mitochondria-mediated apoptosis and autophagy (18). Oxidative stress is recognized as a key contributor to liver aging and the decline in laying performance, disrupting hepatic redox balance, triggering inflammatory responses, lipid metabolism disorders, and fibrosis, as well as impairing ovarian function and reducing egg production (19, 20). As the liver is the central metabolic organ, its dysfunction leads to an overall decrease in the organism's disease resistance. Therefore, alleviating oxidative stress and inhibiting excessive cell death responses is critical strategies to improve liver function.

Although apoptosis and autophagy play protective roles to some extent, their excessive activation under sustained stress can accelerate cellular depletion and organ functional decline (21). In this study, the liver tissues of broiler breeders in the control group without any intervention showed appreciably increased expression of pro-apoptotic factors BAX and Caspase3, decreased expression of the anti-apoptotic factor BCL2, and elevated Beclin1 levels along with an increased LC3-II/LC3-I ratio, indicating simultaneous activation of apoptosis and autophagy. Previous studies have also demonstrated that oxidative stress can regulate the Bax/Bcl-2 axis and Caspase family via ROS accumulation, leading to a coexistent mechanism of autophagy and apoptosis-induced tissue damage (22). However, dietary supplementation with AMK or PAMK exhibited protective effects on the liver. It has been reported that herbal medicines or polysaccharide components can enhance antioxidant capacity, inhibit hepatic oxidative stress, suppress cell apoptosis, and ultimately ameliorate liver injury (23, 24). In evaluating hepatoprotection, the selection of biomarkers is critical. We used ALT, AST, MDA, SOD, CAT, and GSH-Px—well-established indicators of hepatic injury and oxidative status. Studies have shown that these and other hepatic risk markers in predictive models of liver disease, supporting their reliability for assessing hepatic health in animal studies and can complement AMK- or PAMK-based interventions for proactive poultry health management (25). Importantly, supplementation with either AMK or PAMK reversed these pathological changes. Notably, PAMK exhibited more pronounced protective effects, suggesting higher bioactivity than crude AMK. This may be attributed to its higher purity and more specific action on cellular signaling pathways. These findings are consistent with previous studies reporting that crude extracts from Glycyrrhiza uralensis and A. macrocephala improved antioxidant status in poultry (26, 27). Furthermore, previous studies have indicated that crude herbal compound preparations not only offer a cost-effective alternative in practical applications but also exhibit enhanced bioadaptability and broader regulatory potential due to their complex composition (27, 28). Atractylodes macrocephala Koidz. (AMK), a traditional Chinese medicinal herb, possesses a variety of biological functions, including antioxidant, anti-inflammatory, and immunomodulatory effects. Among its active components, PAMK have been identified as major bioactive constituents, exhibiting notable antioxidant, anti-inflammatory, and anti-aging properties in various animal models (29). Plant-derived polysaccharides are recognized as natural immunomodulators that can not only alleviate stress and enhance immune responses and disease resistance in poultry but also help regulate gut microbial balance, thereby mitigating the adverse effects of various stressors encountered during poultry production (30). Preliminary findings have demonstrated that PAMK effectively reduces hepatic oxidative stress and inflammatory responses (31). Notably, increased hepatic Nrf2 protein expression and enhanced antioxidant enzyme activities observed in the PAMK-treated group suggest that its protective effects are closely associated with the Nrf2 signaling pathway. Similar mechanisms have been reported for other natural polysaccharides, such as those derived from Polygonatum sibiricum, and Artemisia annua, which alleviate oxidative damage and inflammatory responses in hepatocytes by activating Nrf2 and suppressing the TLR4/NF-kB or mTOR-associated autophagy pathways (32, 33).

Under oxidative conditions, Nrf2 is released from its cytoplasmic inhibitor Keap1, translocates to the nucleus, and promotes the transcription of antioxidant genes such as HMOX1 and NQO1. In our study, PAMK treatment significantly increased the mRNA expression of Nrf2, CAT, and Msr, while downregulating Keap1. Correspondingly, protein expression levels of HMOX1 and NQO1 were elevated. Previous studies have reported that astaxanthin exerts antioxidant and cytoprotective effects by activating the Nrf2 signaling pathway, thereby alleviating hepatic lipid-metabolic dysregulation (34). Moreover, the partial loss of PAMK-mediated protection in the presence of the Nrf2 inhibitor ML385 provides direct evidence for an Nrf2-dependent mechanism. Interestingly, in the H_2_O_2_-treated group, we observed increased mRNA levels of Nrf2, HMOX, and NQO1, but the corresponding protein levels did not rise proportionally. This discrepancy may be attributed to translation inhibition or enhanced proteasomal degradation of antioxidant proteins induced by oxidative stress. PAMK's ability to normalize both transcript and protein levels suggests that it not only promotes gene transcription but may also stabilize the Nrf2 protein or attenuate proteasomal degradation, thereby restoring functional antioxidant defenses. Collectively, our findings demonstrate that both AMK and PAMK alleviate hepatic oxidative stress and modulate the Nrf2 signaling pathway *in vivo*, with PAMK showing superior efficacy in restoring antioxidant enzyme activity, reducing oxidative damage, and preserving liver histology. These results highlight the potential of PAMK as a more potent and targeted hepatoprotective agent in poultry. It is worth noting that the *in vitro* mechanistic assays in this study were conducted exclusively with PAMK. This choice was based on the *in vivo* evidence that PAMK consistently outperformed crude AMK in mitigating oxidative injury, and thus was prioritized for cellular-level verification. Nevertheless, the absence of AMK in the *in vitro* experiments limits the ability to directly compare their molecular mechanisms. While it is plausible that AMK may also activate the Nrf2 pathway, the magnitude and downstream effects remain to be confirmed. In addition, AMK is a crude extract whose chemical composition, including the relative abundance of polysaccharides, flavonoids, and volatile oils, may vary between batches due to differences in plant growth conditions, harvest time, and extraction efficiency. Such variability could influence biological activity and partially account for inconsistencies between studies. Future research should therefore incorporate chemical profiling of each batch (e.g., HPLC, GC-MS) and adopt standardized extraction and quality control procedures to ensure reproducibility and facilitate cross-study comparisons.

Notably, BCL2 is known to bind to Beclin1 and inhibit the formation of autophagosomes, suggesting that PAMK is involved in the coordinated suppression of cell death pathways (35–37). These results are consistent with studies on other plant-derived polysaccharides. For instance, herbal components such as naringenin and nobiletin may inhibit autophagy activity by regulating multiple pathways and alleviate oxidative stress, thereby exerting protective effects on hepatocytes (38, 39). The cytochrome P450 (CYP450) enzyme system, regulated in part by the pregnane X receptor (PXR), plays a central role in hepatic metabolism of xenobiotics, including herbal compounds. Yi-wen et al. showed that oridonin modulated CYP450 expression in PXR-humanized mice, suggesting that herbal polysaccharides may influence drug metabolism or interact with detoxification pathways (40). This could have implications for PAMK's metabolic processing and its interaction with other feed additives or medications.

From the perspectives of cost-effectiveness and practical application, AMK demonstrates certain advantages as a substitute for PAMK. Although its anti-oxidative, anti-apoptotic and anti-autophagic effects are slightly weaker than those of PAMK, AMK is cheaper and scalable. In a related study, both PAMK and Jiawei Si-jun-zi Decoction effectively substituted antibiotics by modulating the antioxidant system and maintaining intestinal microbial balance under immunosuppressive conditions (41). The high purity of PAMK offers advantages in precisely regulating oxidative stress and immune responses. In contrast, the complex phytochemical composition of AMK may exert similar effects via multi-target synergistic mechanisms, thereby providing dual options for cost-efficient antibiotic alternatives. Although PAMK offers more defined mechanisms and potency, AMK is more economical and accessible, making it a viable option for large-scale application in poultry production. However, variability in AMK composition due to differences in sources and processing methods may impact consistency, highlighting the need for standardization or combination strategies.

With increasing restrictions on the use of antibiotics in poultry farming, the search for safe and effective alternatives has become a research focus (42, 43). Herbal feed additives, known for their natural origin, safety, and lack of residue, have emerged as ideal candidates (44, 45). While PAMK showed superior efficacy due to its well-defined and stable mechanisms, AMK offers an economically viable option with promising practical prospects. The combined or tiered use of PAMK and AMK may warrant further investigation and optimization in poultry farming.

## 5 Conclusion

In conclusion, this study demonstrates that both AMK and PAMK exert significant hepatoprotective effects in laying hens during the peak laying period. PAMK exhibited stronger antioxidant and immunomodulatory activities, likely through activation of the Nrf2 signaling pathway and its associated regulatory networks. These findings highlight the potential of PAMK as a practical and safe feed additive for improving liver health and productivity in poultry. Future research should focus on dose optimization, evaluation in diverse poultry species and stress models, and deeper exploration of its molecular targets, including crosstalk with the PI3K/Akt and cytochrome P450 pathways, to further support its application in sustainable poultry production.

## Data Availability

The datasets used in this study have been uploaded to Figshare and can be accessed at the following URL: https://doi.org/10.6084/m9.figshare.29377238.v1. The Supplementary material is the experimental supplementary material.
