# Supplementary material for: Comparative hepatoprotective effects of AMK and PAMK via Nrf2 signaling in broiler breeders
Source: Front Vet Sci. 2025 Sep 23;12:1672417. doi: 10.3389/fvets.2025.1672417 (PMC12502850; doi:10.3389/fvets.2025.1672417)
Supplement: Supplementary file 1 [file Data_Sheet_1.pdf]

Supplementary Material

1 SUPPLEMENTARY MATERIAL

The data involved in this study has been uploaded to Figshare https://doi.org/10.6084/m9.figshare.29377238l, available for reading and reference by readers.

2 PRELIMINARY EXPERIMENT

ROS fluorescence staining was used to assess the impact of H_2O_2 on chicken embryo hepatocytes and to determine the optimal concentration of PAMK. As shown in Figure S1, both 60 μg/mL and 120 μg/mL PAMK significantly alleviated H_2O_2-induced oxidative stress (P ¡ 0.05). However, 120 μg/mL elicited slight cytotoxicity; therefore, 60 μg/mL was selected as the optimal concentration for subsequent experiments.

2.1 Figures

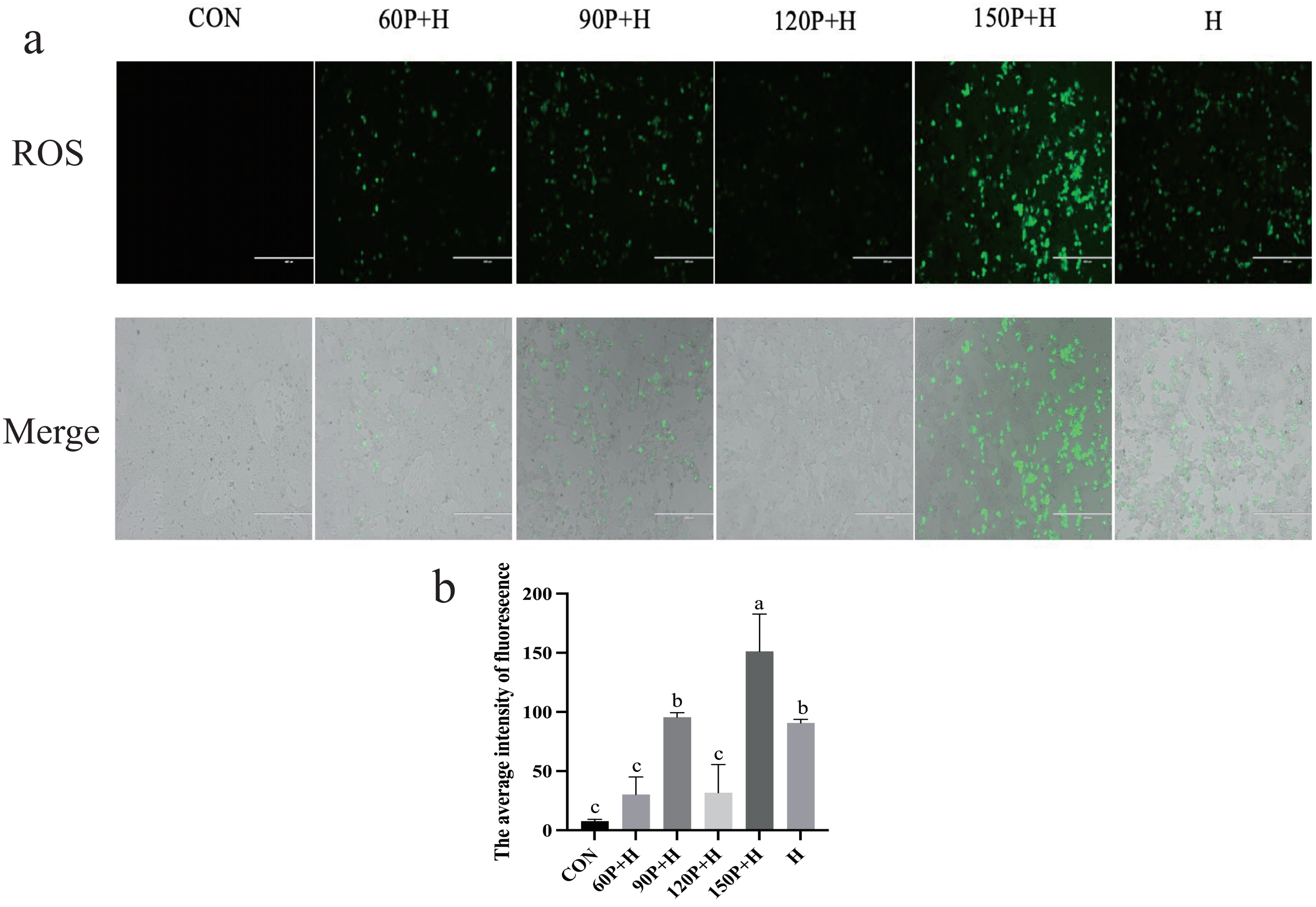

Figure S1. Validation of the optimal PAMK concentration. (a) ROS fluorescence. (b) Mean fluorescence intensity.
